# RNA-Seq and microarray analysis of the *Xenopus* inner ear transcriptome discloses orthologous OMIM^®^ genes for hereditary disorders of hearing and balance

**DOI:** 10.1186/s13104-015-1485-1

**Published:** 2015-11-18

**Authors:** Daniel Ramírez-Gordillo, TuShun R. Powers, Jennifer C. van Velkinburgh, Casilda Trujillo-Provencio, Faye Schilkey, Elba E. Serrano

**Affiliations:** Biology Department, New Mexico State University (NMSU), Las Cruces, NM 88003 USA; National Center for Genome Resources (NCGR), Santa Fe, NM 87505 USA

**Keywords:** Deafness, Genomics, Mechanosensory hair cell, Vestibular, *X. laevis*, *X. tropicalis*

## Abstract

**Background:**

Auditory and vestibular disorders are prevalent sensory disabilities caused by genetic and environmental (noise, trauma, chemicals) factors that often damage mechanosensory hair cells of the inner ear. Development of treatments for inner ear disorders of hearing and balance relies on the use of animal models such as fish, amphibians, reptiles, birds, and non-human mammals. Here, we aimed to augment the utility of the genus *Xenopus* for uncovering genetic mechanisms essential for the maintenance of inner ear structure and function.

**Results:**

Using *Affymetrix* GeneChip^®^*X. laevis* Genome 2.0 Arrays and Illumina-Solexa sequencing methods, we determined that the transcriptional profile of the *Xenopuslaevis* inner ear comprises hundreds of genes that are orthologous to OMIM^®^ genes implicated in deafness and vestibular disorders in humans. Analysis of genes that mapped to both technologies demonstrated that, with our methods, a combination of microarray and RNA-Seq detected expression of more genes than either platform alone.

**Conclusions:**

As part of this study we identified candidate scaffold regions of the *Xenopus tropicalis* genome that can be used to investigate hearing and balance using genetic and informatics procedures that are available through the National *Xenopus* Resource (NXR), and the open access data repository, *Xenbase*. The results and approaches presented here expand the viability of *Xenopus* as an animal model for inner ear research.

**Electronic supplementary material:**

The online version of this article (doi:10.1186/s13104-015-1485-1) contains supplementary material, which is available to authorized users.

## Background

The vertebrate inner ear serves a unique dual function as a receptor for the senses of hearing and balance. The inner ear’s ability to detect sound and maintain spatial orientation is vital to the survival of an organism. In humans and other vertebrates, disorders of hearing and balance can lead to disabilities that can reduce the lifespan, and in the case of humans, severely compromise the quality of life. It is estimated that approximately 1 in 20 Americans are hard of hearing or are functionally deaf, and that 35 % of adults ages 40 and older (69 million Americans) have experienced vestibular dysfunction during some period in their lives [1, 2]. Vestibular and auditory disorders are frequently caused by damage to the mechanosensory hair cells of the inner ear or to the afferent and efferent neurons of the eighth cranial nerve [2, 3]. The number of affected individuals who experience difficulties with hearing or balance is expected to increase as the population ages [2]. In order to address this growing global health challenge, it is crucial to understand the mechanisms that underlie the senses of hearing and balance, and especially to identify the genetic basis of acoustic-vestibular health and dysfunction [3, 4].

Human inner ears are typically not a feasible choice for genetic studies due to the invasive nature of the technical approaches, and the frequent need to terminate animal life as part of the experimental design. Consequently, non-human animal models are the foundation for acoustico-vestibular research [5, 6]. When choosing an organism to study the mechanisms of hearing and balance with the goal of addressing human health, it is advisable to select an organism whose inner ear resembles that of humans. The amphibian inner ear is similar to the human inner ear, with the notable exception that sound reception is distributed among three sensory organs instead of the solitary cochlea [7–9]. Like humans, and many other mammals, amphibians are capable of vocal communication. However, unlike mammals, amphibians can regenerate damaged hair cells and their sensory epithelia have the capacity for the perpetual production of new hair cells throughout animal life [5, 6]. These characteristics confer an additional benefit to the use of animals from this class for research that aims to discover mechanisms that can restore function to damaged inner ears.

*Xenopus*, the African clawed frog, is an amphibian native to Sub-Saharan Africa that has become an increasingly popular subject for inner ear investigations over the past decade, especially for research that addresses embryonic placode and larval organ formation [7, 10, 11]. The genus *Xenopus* comprises about 15 species of frogs; the two most common research species are *Xenopus laevis* and *Xenopus tropicalis* both of which have long been used in medical and scientific investigations [12]. An established model for developmental studies of embryogenesis, *Xenopus*’ contributions to medicine date to the 1930s, when pregnancy was confirmed by the induction of ovulation in female *Xenopus* injected with the urine of human females [13]. Almost a century of research has shown conservation of cellular, developmental, and genomic organization between *Xenopus* and mammals, and established its utility for investigations of organ and tissue repair due to *Xenopus’* documented regenerative capacity [14, 15].

Understanding of the genetic underpinnings of hearing and balance common to *Xenopus* and humans can be furthered by using high throughput approaches to profile the cadre of genes whose collective expression is required in a functioning inner ear organ [16, 17]. To this end we used bioinformatics tools, in combination with microarrays and RNA-Seq, to determine whether Online Mendelian Inheritance in Man^®^ (OMIM^®^) orthologues for hearing and balance are expressed in the *Xenopus* inner ear. Our experimental approach also was designed to permit comparison of the capacity of these two technologies to detect candidate OMIM^®^ orthologues in *Xenopus* transcriptomes.

We began by mining the OMIM^®^ database to assemble a list of OMIM^®^ genes associated with both deafness and vestibular disorders, with deafness, or with vestibular disorders. Informatics and curation approaches were used to identify candidate orthologues to OMIM^®^ deafness and vestibular disorder genes among the *Xenopus laevis* Probe Set Identifiers (Xl-PSIDs) on the Affymetrix Genechip^®^*X. laevis* Genome 2.0 Array, and among the scaffolds of the *X. tropicalis* Genome Assembly v.2. Gene expression was evaluated for candidate OMIM^®^ orthologues that met threshold criteria established for the two technologies. Our results suggest that orthologues for 259 (74 %) of the human OMIM^®^ genes associated with auditory and vestibular function are expressed in the *Xenopus* inner ear at the animal age examined in this study. When we undertook a detailed expression analysis of the 190 OMIM^®^ deafness and vestibular disorder genes that could be mapped to both the Xl-PSIDs on the microarray and *X. tropicalis* genome scaffolds, we found that a combination of both approaches uncovered expression of more candidate OMIM^®^ orthologues than either method alone. Taken together, the results and the approaches presented here enhance the relevance of the genus *Xenopus* for investigations of the sensory systems for hearing and balance.

## Methods

### Inner ear RNA

Larval *Xenopus laevis* stages 56–58 with fully formed inner ears [7, 10, 18] were obtained from Nasco (Fort Atkinson, WI, USA). Specimens were euthanized in a 2 % solution of ethyl 3-aminobenzoate methanesulfonate salt before aseptic removal of the inner ear tissue. Protocols were approved by the Institutional Animal Care and Use Committee (IACUC) of New Mexico State University.

Biological samples for microarray and RNA-Seq analysis comprised 16–20 inner ears (8–10 animals) per RNA sample (3 for microarray; 1 for RNA seq). Inner ear RNA was isolated with the Qiagen^®^ RNeasy^®^ Mini Kit according to established procedures [19]. RNA quality was assessed using the Agilent 2100 Bioanalyzer. Experimental samples with a minimum RNA integrity number (RIN) of 9.0 were shipped to the National Center for Genome Resources, (NCGR; Santa Fe, NM, USA) for Illumina-Solexa sequencing or to the Massachusetts Institute of Technology (MIT; Cambridge, MA, USA) BioMicro Center for microarray analysis with the Affymetrix GeneChip^®^*X. laevis* Genome 2.0 Array.

### Profiling inner ear RNA with Illumina-Solexa sequencing and Affymetrix GeneChip^®^*Xenopus laevis* Genome 2.0 Array

RNA-sequencing was completed using the Illumina-Solexa platform for sequencing by synthesis. As recommended by the manufacturer, short-insert paired end (SIPE) libraries were prepared from total RNA according to Illumina’s mRNA-Seq Sample Prep Protocol v2.0 (Illumina, San Diego, CA, USA). The resultant double-stranded cDNA concentration was measured on a NanoDrop spectrophotometer, and size and purity were determined on the 2100 Bioanalyzer using a DNA 1000 Nano kit. The cDNA libraries were cluster amplified on Illumina flowcells, sequenced on the GAII Sequencer as 36-cycle single-end reads, and processed using Illumina software v1.0. The RNA sample produced 28,764,261 Illumina reads with an average quality score above Q30. The reads were aligned to the *X. tropicalis* genome (UCSC XenTro3; JGI 4.1) using the algorithm for genomic mapping and alignment program (GMAP) and Alpheus^®^ Sequence Variant Detection System v3.1 [20]. The GMAP program was used by Alpheus^®^ to align high quality reads containing bases with a PHRED score ≥20 with default parameter settings, except base pair mismatches was set to 2 (based on read length). Alpheus^®^ was used to normalize reads per million (RPM). The total number of reads mapped to each of the OMIM^®^ genes were log_2_ transformed. Fifty-one percent of reads aligned to the *X. tropicalis* reference genome. The microarray procedures and data were processed as previously described [17]. Data from three biological replicates were preprocessed using Gene Chip robust multichip averaging (GCRMA) to generate a log_2_ transformed intensity measure for each Xl-PSID.

The original data analyzed in this publication have been deposited in NCBI’s Gene Expression Omnibus and are accessible through GEO Series accession numbers GSE69701 (RNA-Seq) and GSE73829 (microarray).

### Identification of OMIM deafness and vestibular disorder genes

The OMIM^®^ database was regularly accessed and manually inspected (most recently, May 2015) to identify a list of 299 HUGO Gene Nomenclature Committee (HGNC) symbols pertaining to deafness by using the query term “deafness” and limiting the results to known sequences and phenotypes to search for syndromic and non-syndromic deafness genes in humans [21]. Using the same criteria described above, but using the query term “vestibular”, a list of 123 HGNC symbols was obtained for genes associated with vestibular disorders [21]. The OMIM^®^ deafness and vestibular disorder genes symbols were analyzed to determine genes uniquely associated with deafness only, vestibular disorder only or dual phenotype (deafness and vestibular disorders). Human protein sequences were obtained for 279 of the 299 deafness HGNC symbols and 120 of the 123 vestibular disorder HGNC symbols as described in Powers et al. [17]. Genes uniquely associated with deafness, vestibular disorders, or dual phenotype (deafness and vestibular disorders) were identified by comparing the deafness OMIM gene list with the vestibular disorder OMIM^®^ gene list (Table 1; Additional file 1).

**Table 1:** Expression analysis for OMIM^®^ genes that aligned to *X. tropicalis* genome scaffolds and *X. laevis* PSIDs

Category	Deafness	Vestibular	Both	Total
OMIM^®^ genes	229	53	70	352
*X. tropicalis* genome				
OMIM^®^ genes that mapped to *Xt* genome scaffolds and met alignment criteria	204	51	67	322^a^
OMIM^®^ genes that mapped to *Xt* genome scaffolds, met alignment criteria, and met RNA-Seq expression criteria	161	29	51	241
Affymetrix Xl-PSIDs				
OMIM^®^ genes that mapped to Xl-PSID genes that met alignment criteria	154	40	40	234^a^
OMIM^®^ genes that mapped to Xl-PSID genes that met alignment and expression criteria	96	12	18	126^b^

^a^190 OMIM genes met the alignment criteria for both technologies (see Fig. 2)

^b^This group includes 18 OMIM^®^ genes that were not detected by RNA-Seq

### Analysis of OMIM^®^ deafness and vestibular disorder orthologous genes in the *Xenopus* inner ear transcriptome

**Fig. 1:**
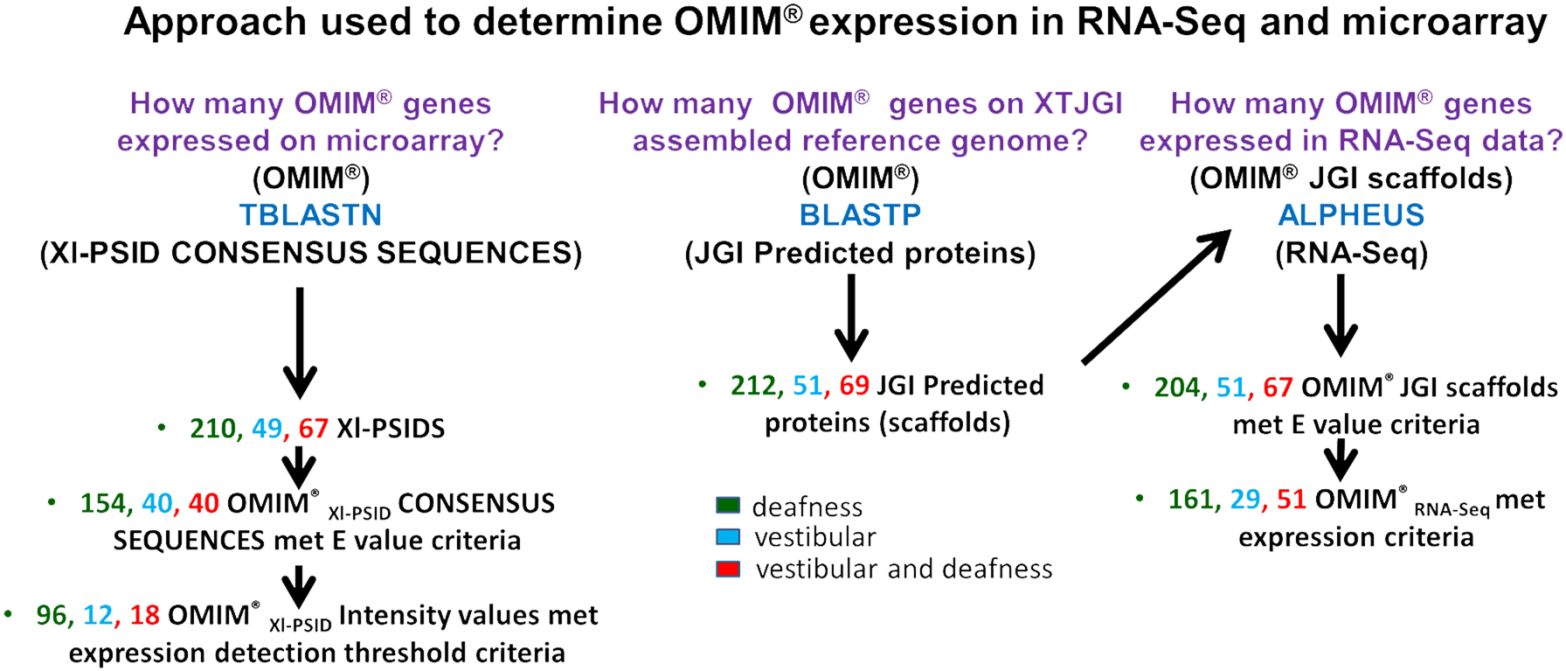
Experimental strategy for determining the expression of OMIM^®^ orthologues for deafness and vestibular disorder genes in the *Xenopus* inner ear transcriptome with Illumina-Solexa (RNA-Seq) and *Affymetrix* microarray methods. A comprehensive list of OMIM^®^ genes for deafness and vestibular genes was manually curated and used to map OMIM^®^ sequences to the Xl-PSIDs on the *Affymetrix GeneChip* ^*®*^ *X. laevis Genome Array*, and to the JGI *Xenopus* reference genome scaffolds. *Xenopus* inner ear RNA was used in microarray hybridization reactions with the *Affymetrix GeneChip* ^*®*^. The hybridization data were analyzed to retrieve intensity values from target Xl-PSIDs that had met alignment criteria for OMIM^®^ orthologues for deafness and vestibular disorder genes. The Alpheus^®^ program was implemented to map inner ear RNA-Seq reads to the JGI *Xenopus* reference genome scaffolds and the RNA-Seq alignment data were analyzed to retrieve target scaffold regions that had met alignment criteria for OMIM^®^ orthologues for deafness and vestibular disorder genes. As part of the analysis, the data were separated into three OMIM^®^ phenotype categories: deafness only; vestibular disorder only; and both deafness and vestibular disorder. When expression criteria were applied to both datasets, RNA-Seq methods detected expression of more OMIM^®^ orthologues for deafness and vestibular disorder genes in the *Xenopus* inner ear (241) than were detected by microarray methods (126)

Figure 1 summarizes the approach used to determine expression of OMIM^®^ orthologues in RNA-Seq and microarray data. Human protein sequences corresponding to the HGNC symbols were retrieved from the Ensembl website (release 79). Standalone BLAST was used to map the human protein sequences to the *X. laevisAffymetrix* consensus sequences (provided by the manufacturer) and the *X. tropicalis* predicted proteins. The consensus sequences (15,491) were downloaded directly from *Affymetrix;* predicted protein sequences (27,916) were downloaded from the Joint Genome Institute (JGI) genome portal. The scaffold number and coordinates for each of the OMIM^®^ genes were also acquired from the JGI genome portal. For RNA-Seq, the BLASTP alignments of the Ensembl human protein sequences to the JGI *X. tropicalis* predicted proteins provided the predicted protein designation number for *Xenopus* orthologues of OMIM deafness and vestibular disorder genes. The protein designation number was then entered into the JGI *Xenopus tropicalis* v4.1 genome portal to obtain the corresponding scaffold number and coordinates. The scaffold number and coordinates were then entered into the Alpheus^®^ Sequence Variant detection software to obtain the number of RNA-Seq reads for each corresponding OMIM gene.

For microarray data, the TBLASTN alignments of the Ensembl human protein sequences to the Affymetrix *X. laevis* GeneChip^®^ consensus sequences retrieved a list of microarray Xl-PSIDs whose intensity values were used for data analysis. The protocol and criteria for the BLAST alignments were identical to those previously reported in an open source publication; alignments that met a threshold E-value criterion of 10^−14^ were used for downstream analysis (H, high similarity (E ≤ 10^−100^); M, moderate similarity (E  =  10^−99^ to 10^−50^); W, weak similarity (E  =  10^−49^ to 10^−15^) [17]. Some of the OMIM^®^ genes mapped to multiple predicted proteins or to multiple Xl-PSIDs that met the detection criteria. To avoid counting the same OMIM^®^ gene twice we chose the predicted protein or Xl-PSID with the highest E value (Additional file 1). Regression analysis was undertaken to determine correlation between RNA-Seq reads and GCRMA value (microarray) for the same genes that met expression threshold criteria for both technologies. The regression was completed in the Excel data analysis tool using the values for orthologues that met the expression criteria for both technologies.

## Results

### Identification of *X. tropicalis* scaffolds for RNA-Seq analysis

BLASTP (standalone BLAST version 2.2.27+) was used to align the protein sequences of deafness and vestibular disorder genes acquired from OMIM^®^ (deafness, n = 281; vestibular disorder, n = 120) to *X. tropicalis* predicted protein sequences downloaded from JGI *X. tropicalis* genome version 4.1 [22]. This process resulted in 281 alignments for deafness and 120 for vestibular disorders. After removal of the alignments that fell below the E value criteria of 10^−14^ or greater [17], the number of OMIM^®^ genes that mapped to proteins corresponding to *X. tropicalis* scaffolds was reduced to 271 for deafness and 118 for vestibular disorders (Table 1; Fig. 1).

### Detection of *Xenopus* OMIM orthologue expression with Illumina-Solexa RNA-Seq

The Alpheus^®^ software identified RNA-Seq reads that aligned to *X. tropicalis* scaffolds for deafness and vestibular disorders that met Illumina’s criteria of a minimum of 10 reads [23]. Further analysis revealed that 222 deafness and 86 vestibular disorder alignments for proteins with corresponding *X. tropicalis* scaffolds met the detection threshold criteria for RNA-Seq expression (data not shown). We noted that some OMIM^®^ genes aligned to the same *X. tropicalis* scaffold for predicted proteins. To avoid duplicate analysis, we analyzed RNA-Seq expression for the OMIM^®^ genes using the protein alignment with the highest E value that exceeded, or was equal to, the detection criteria. This procedure reduced the OMIM^®^ gene lists to 212 and 80 for deafness and vestibular disorders, respectively (Table 1; Fig. 1).

### Detection of *Xenopus* OMIM^®^ orthologue expression with Affymetrix microarray

TBLASTN (standalone BLAST version 2.2.27+) was used to align the protein sequences of the deafness and vestibular disorder genes acquired through OMIM^®^ (deafness, n = 281; vestibular disorder, n = 120) to consensus sequences from the Affymetrix *X. laevis* GeneChip^®^ version 2. Analysis showed 277 alignments for deafness and 116 for vestibular disorders. After removal of the alignments that fell below the E value criteria, the number was reduced to 194 for deafness and 80 for vestibular disorders. We then imposed an expression threshold criterion for GCRMA values of 4 or higher [17], thereby identifying 130 deafness and 42 vestibular disorders OMIM^®^ genes that met the expression threshold criterion. Some of the OMIM^®^ genes mapped to multiple Xl-PSIDs that met the expression criteria. To avoid counting the OMIM^®^ gene twice we chose only the OMIM^®^ gene that mapped to the Xl-PSID with the highest E value. This procedure reduced the OMIM^®^ detection list to 114 OMIM^®^ deafness genes and 30 OMIM^®^ vestibular disorder genes (Table 1; Fig. 1).

### Comparison of RNA-Seq and microarray detection of OMIM deafness and vestibular disorder orthologues

**Fig. 2:**
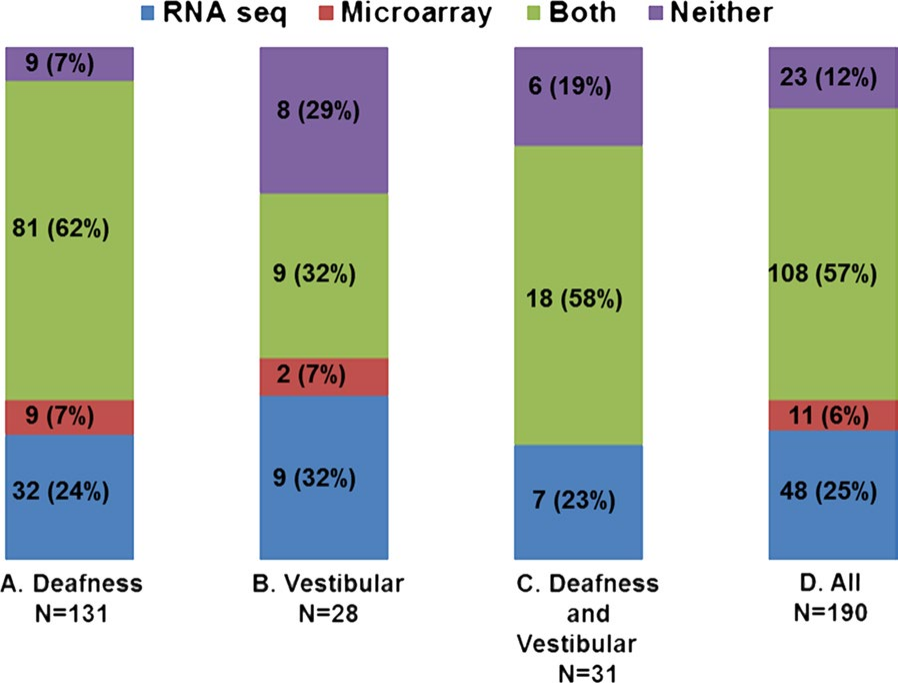
Comparison of Illumina-Solexa (RNA-Seq) and *Affymetrix* microarray methods for detection of *Xenopus* orthologues for OMIM^®^ deafness and vestibular disorder genes.* Bar graphs* compare detection data for 131 deafness only OMIM^®^ genes (**a**), 28 vestibular disorder only OMIM^®^ genes (**b**), 31 OMIM^®^ genes that are associated with both vestibular and deafness phenotypes (**c**) and all 190 OMIM^®^ genes (**d**) that met *alignment* criteria for both microarray and RNA-Seq analysis. Both technologies met *expression* criteria for 108 (57 %) of the 190 OMIM^®^ genes. RNA-Seq met expression criteria for 48 (25 %) additional OMIM^®^ genes not detected by microarray. Eleven (6 %) genes were detected by microarray methods only. Expression criteria were not met with either technology for 23 (12 %) of the 190 *Xenopus* OMIM^®^ orthologues that met alignment criteria

A common set of 190 *Xenopus* OMIM^®^ orthologues was identifiable on the *X. tropicalis* scaffold and among the Xl-PSIDs on the Affymetrix Genechip (Fig. 2). When we compared the capacity of the two technologies to detect OMIM^®^ genes, we noted that RNA-Seq detected 48 OMIM^®^ genes that were not detected by the microarray (Fig. 2). However, eleven OMIM^®^ genes met the expression criteria for the microarray but were not detected by RNA-Seq. Altogether, 108 OMIM^®^ genes were detected by both technologies and there were 23 OMIM^®^ genes that did not meet the detection limit for either technology (Fig. 2).

### Correlation between RNA-Seq read-based intensity values and microarray Xl-PSID intensity values for *Xenopus* OMIM^®^ orthologues

**Fig. 3:**
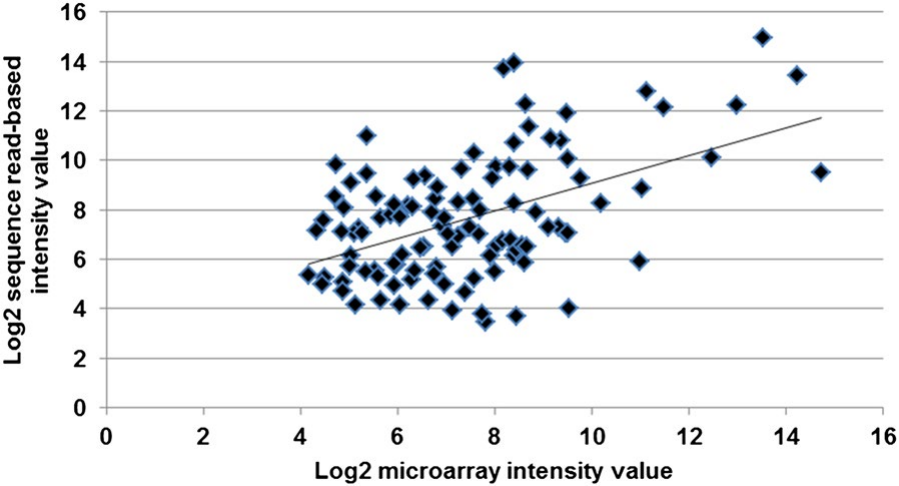
Correlation plot of *Affymetrix* microarray and Illumina-Solexa (RNA-Seq) measurements of OMIM^®^ gene expression. Data (log_2_) from 108 *Xenopus* orthologues for OMIM^®^ deafness and vestibular disorder genes that met expression criteria for both technologies are plotted in the figure (X-axis, microarray GCRMA intensity values; Y- axis, RNA-Seq read based intensity value). A modest correlation was detected between RNA-Seq reads and microarray intensity values in this analysis (R = 0.49, R^2^ = 0.24, P value <0.5)

We were interested in determining the extent of the correlation between the RNA-Seq and microarray detection values for individual OMIM^®^ genes whose expression could be evaluated by the two technologies. Figure 3 shows a correlation plot of the log_2_ expression levels (in arbitrary units) for OMIM^®^ genes that met the detection criteria for both technologies. A regression analysis established a moderate correlation between microarray and RNA-Seq (R = 0.49; R^2^ = 0.24; P < 0.5).

### OMIM^®^ genes grouped into single or dual phenotypes

Table 1 shows the number of OMIM^®^ genes that were associated with both deafness and vestibular disorders as well those associated with only one of two disorders. The majority of the OMIM^®^ genes (229) were associated only with deafness, 53 OMIM^®^ genes were associated only with vestibular disorders, and 70 OMIM^®^ genes were identified as associated with both phenotypes (Table 1). When comparing RNA-Seq and microarray, RNA-Seq detected 70 % of the deafness (only) genes, 55 % of the vestibular disorder (only) genes, and 73 % of the dual phenotype genes (Table 1; Fig. 1). In contrast, the microarray approach detected 42 % of the deafness (only) genes, 23 % of the vestibular disorder (only) genes, and 26 % of the dual phenotype genes (Table 1; Fig. 1).

## Discussion

*Xenopus* is a well-established model organism for investigations of early embryogenesis and development [18, 24, 25]. The last decade has witnessed a rapid expansion in the *Xenopus* experimental repertoire through development of methods for *Xenopus* transgenesis and genome editing (CRISP-R; TALEN), as well as a robust and comprehensive online database (*Xenbase),* a central national laboratory for production of transgenic animal stocks and research training (NXR), and *Xenopus* genome assemblies [25–29]. The development of algorithms, such as Unveiling RNA Sample Annotation (URSA), and Phylogenetically-Informed Annotation (PIA) are predicted to accelerate annotation of *Xenopus* genes at a faster and more precise rate than traditional manual annotation, thereby increasing the impact of high throughput experiments undertaken with *Xenopus* [30, 31]. Here we have shown that a combination of data mining and experimental approaches can be implemented to evaluate and expand the utility of *Xenopus* for genetic studies of human hereditary disorders such as those that affect the senses of hearing and balance. Our methods identified several hundred *Xenopus* OMIM^®^ orthologues for deafness and vestibular disorders that met the criteria for RNA-Seq (241) and microarray (126) expression (Fig. 1; Table 1). These findings confirm the suitability of *Xenopus* as a model organism for genetic and functional analysis of OMIM^®^ orthologues that are predicted to affect auditory and vestibular sensation in humans. Moreover, the identification of scaffolds for OMIM^®^ orthologues on the *X. tropicalis* genome will accelerate genetic analysis because this species offers the advantage of a shorter generation time, a smaller diploid genome, and a more detailed reference assembly than its relative, *X. laevis* [12].

Our analysis also showed that 70 OMIM^®^ genes are associated with both deafness and vestibular disorders, as expected from the similarities between the mechanosensory processes of hearing and balance and common features of the sensory epithelia (Table 1; Figs. 1, 2). In fact, diseases that affect the inner ear can disrupt both hearing and balance, often due to damage to the mechanosensory hair cells that function as cellular receptors for both sensory modalities [16, 32]. Mutations in these 70 OMIM^®^ genes potentially could cause complications for both hearing and balance. In contrast, 229 genes were associated with deafness only and 53 genes were associated with vestibular disorders only, suggesting that damage to any of these genes could result in complications in hearing or balance but not both (Table 1; Figs. 1, 2). The specificity of some genes for auditory or for vestibular disorders may arise from functional and structural differences that are required for sensory endorgans to accomplish their specialized task for sense reception and frequency discrimination [2, 9]. For example, the spiral shaped cochlea is the major organ involved in the hearing process, while the elongated semicircular canals, and sack-like utricle and sacculus with their overlying otolith crystals, function specifically in the vestibular system in humans [33]. Another explanation is that far less is known about the vestibular system in comparison to hearing; as more is learned about the vestibular system, we may discover that other genes that affect hearing also affect balance [3].

We observed that Illumina Solexa RNA-Seq methods detected expression of more *Xenopus* orthologues for deafness and vestibular disorder genes than the Affymetrix microarrays (Table 1; Figs. 1, 2). These findings are in agreement with studies showing that RNA-Seq technologies offer advantages over microarray technology, such as a greater sensitivity in the detection of gene expression, especially for low abundance transcripts [34–36]. Microarrays are an inherently biased analysis platform because they are optimized to evaluate expression of the subset of genes represented in the probe set. However, it is noteworthy that some deafness and vestibular disorder genes were detected by the microarray but not by RNA-Seq. This indicates that genes may be overlooked if only one technology is used, suggesting that the two technologies can complement each other and that a combination of both approaches can provide a more complete analysis of gene expression than either method alone [37–39]. It is intriguing that regression analysis showed moderate correlation between RNA-Seq and microarray for the genes that met expression criteria for both technologies (Fig. 3). For example, some genes detected on the microarray were scored with high GCRMA intensity values (high expression), but with low corresponding RNA-Seq read counts, and the converse was also observed. One possible explanation for this finding is that isoforms of a gene differ by only a few bases. It is also possible that different isoforms of a gene hybridized to the same probe in the microarray, resulting in a higher GCRMA value [40]. On the other hand, low GCRMA values may be the result of poor hybridization of the microarray, which is a known limitation of this technology.

## Conclusion

Here we have shown that by combining data mining and transcriptional profiling, it is possible to identify expression of a cadre of *Xenopus* orthologues for OMIM^®^ genes associated with hereditary human disorders, such as hearing and balance. We also have identified candidate regions of the *X. tropicalis* genome that can be used as targets to develop *Xenopus* models for investigations of human deafness and vestibular dysfunction. Many of the OMIM^®^ orthologues whose expression we have detected have not been the subject of detailed mechanistic or genetic studies in the inner ear of *Xenopus* or other animals. We propose that our experimental approach (Fig. 1) can be implemented by investigators interested in exploring the potential of *Xenopus* for investigations of other human hereditary diseases and disorders.

## Additional file

RNA-Seq and microarray data have been deposited to the Gene Expression Omnibus (GEO) repository under accession numbers GSE69701 (RNA-Seq) and GSE73829 (microarray).

10.1186/s13104-015-1485-1 Master table for *Xenopus* OMIM auditory and vestibular genes. This spreadsheet contains the list of OMIM^®^ deafness and vestibular disorder genes, mappings to the JGI predicted proteins and Xl-PSID’S (E-value for alignment, E-value for expression, scaffold number and coordinates), RNA-Seq reads associated with each OMIM^®^ gene and microarray GCRMA value for each OMIM^®^ gene. The original data discussed in this publication have been deposited in NCBI’s Gene Expression Omnibus and can be accessed under GEO Series accession numbers GSE69701 (RNA-Seq) and GSE73829 (microarray).

## Abbreviations

GCRMA: Gene Chip robust multichip averaging
NXR: National *Xenopus* Resource
OMIM^®^: Online Mendelian Inheritance in Man
Xl: *Xenopus laevis*
Xl-PSID: *Xenopus laevis* probe set ID
Xt: *Xenopus tropicalis*
